# Enhancers Improve the AID-Induced Hypermutation in Episomal Vector for Antibody Affinity Maturation in Mammalian Cell Display

**DOI:** 10.3390/antib7040042

**Published:** 2018-12-13

**Authors:** Chuan Chen, Jie Wang, Yun Zhao, Shaopeng Chen, Zhishang Hu, Long Chen, Haiying Hang

**Affiliations:** 1 Key Laboratory for Protein and Peptide Pharmaceuticals, National Laboratory of Biomacromolecules, Institute of Biophysics, Chinese Academy of Sciences, Beijing 100101, China; ChenChuan299792458@gmail.com (C.C.); chinabzwdwangjie@163.com (J.W.); super@moon.ibp.ac.cn (Y.Z.); 2 University of Chinese Academy of Sciences, No.19(A) Yuquan Road, Shijingshan District, Beijing 100049, China; 3 Hefei Institutes of Physical Science, Chinese Academy of Sciences, Institute of Technical Biology and Agriculture Engineering, 350 Shushanhu Road, Hefei 230031, Anhui, China; spchen035@ipp.ac.cn; 4 National Institute of Metrology, No.18, Bei San Huan Dong Lu, Chaoyang Dist, Beijing 100101, China; huz@nim.ac.cn; 5 Department of Civil and Environmental Engineering, Northeastern University, Boston, MA 02115, USA

**Keywords:** activation-induced cytidine deaminase (AID), somatic hypermutation (SHM), episomal vector, enhancer

## Abstract

The induction of somatic hypermutation (SHM) in various cell lines by activation-induced cytidine deaminase (AID) has been used in protein-directed selection, especially in antibody affinity maturation. Several antibody affinity maturation systems based on mammalian cells have been developed in recent years, i.e., 293T, H1299, Raji and CHO cells. However, the efficiency of in vitro AID-induced hypermutation is low, restricting the application of such systems. In this study, we examined the role of Ig and Ek enhancers in enhancing SHM in the episomal vector pCEP4 that expresses an anti-high mobility group box 1 (HMGB1) full-length antibody. The plasmid containing the two enhancers exhibited two-fold improvement of mutation rate over pCEP4 in an AID expression H1299 cell line (H1299-AID). With the engineered episomal vector, we improved the affinity of this antibody in H1299-AID cells by 20-fold.

## 1. Introduction

Antibodies have become one of the most important protein molecules for therapy and diagnosis. However, oftentimes, antibodies obtained from hybridoma or antibody libraries exhibit low affinity [1,2], which is insufficient for use in diagnosis and medical treatment. To this end, various antibody display technologies have been developed for antibody affinity maturation, such as phage display [3,4], bacteria display [5,6,7], yeast display [8,9], and mammalian cell display [10,11,12,13]. Among all these techniques, the mammalian cell display platform has the advantages of protein expression, more advanced peptide folding and post-translational modifications [14,15,16]. To generate an antibody library in mammalian cells, three main steps are required. 1. Cloning the antibody mutants into mammalian cell expression vectors to generate an antibody library in bacteria. 2. Purifying the library plasmids and transfect the plasmids into mammalian cells. 3. Antibiotic selection to generate stable cells with antibodies displayed on the cell surface. There are two kinds of mammalian cell expression vectors (chromosome-integrating [17] and episomal vectors [10,16,18]) which can be used to develop libraries. In chromosome-integrating vector systems, cells without plasmids inserted into their genome will be killed by antibiotics during the antibiotic selection process. Due to the low efficiency of genome integration (i.e., ≤1%) [19], only small size libraries (10^6^ range) can be generated using chromosome-integrating plasmids in mammalian cells. In the system using episomal vectors, the integration of the antibody-expression vectors into cell genome is not required. However, low transfection efficiency should be chosen to generate cells with low copy number plasmids for expression within the cell [16,20,21,22]. Similar to chromosome-integrating vectors, antibiotic selection also needed to generate stable library cells, and most of the cells will be killed by the antibiotic. The antibody libraries generated by episomal vectors are also small.

Activation-induced cytidine deaminase (AID) enzyme deaminates cytidine residues in IgG genes to initiate somatic hypermutation in both B cells and non-B cells [12,23]. It has been used to generate antibody libraries in mammalian cells [13,16,23,24]. Coupling mammalian cell display and AID-induced somatic hypermutation (SHM), several groups have developed their own antibody selection [25,26] and affinity maturation systems [10,12,13,16,27,28]. However, the rate of AID-induced SHM is less than10^−5^ bp per generation in vitro [12,23], and is over 100-fold lower than the centroblast stage of B cell differentiation (≈10^−3^ bp per generation) [16]. Thus, improving the rate of AID-induced SHM in vitro has great value in mammalian cell display technologies.

It has been previously reported that AID could mutate the target antibody gene encoded in an episomal vector pCEP4 (Invitrogen, Grand Island, NY, USA) in mammalian cells, and significant antibody affinity improvement was achieved in these pCEP4 vector-based systems [16,29]. Unlike regular vectors, pCEP4 can stably replicate and be maintained within mammalian cells as episomes outside of chromosomes. This confers a higher level of transcription of the desired antibody gene than integrated vectors [13,30]. Higher level transcription of the interested antibody gene can generate a higher AID-induced mutation rate [31]. It is widely known that enhancer (a short (50–1500 bp) region of DNA) can bind with specific transcription factors to enhance the transcription of nearby genes [32,33]. Thus, increasing the frequency of AID-induced SHM to nearby transcribed genes [34,35,36,37,38]. We hypothesize that, by placing an enhancer in the pCEP4 vector, higher degree of AID-induced SHM to the target gene on pCEP4 should be obtained. In this work, two different enhancers which were identified from the major introns of the mouse immunoglobulin heavy (IgH) loci (Ig enhancer) [39] and x light chain loci (Ek enhancer) [40] were chosen for the experiments. We tested the mutation rates of a targeted GFP gene sequence in pCEP4 vectors containing one or two different enhancers. The plasmid with two enhancers had two-fold AID-induced SHM than the original pCEP4 vector. Furthermore, we used this system to mature an antibody against high mobility group box 1 (HMGB1) protein [41,42,43,44,45]. Here we chose to mature a full-length anti-HMGB1 antibody (3B1) [46]. Using the engineered enhancer based episomal vector, we hereby improved the affinity of this antibody by 20-fold, demonstrating usefulness of enhancers in antibody affinity maturation.

## 2. Materials and Methods

### 2.1. Plasmids Construction

To construct pPuro-hAID plasmid, the hygromycin resistant gene in pCDNA3.1/Hygro(+) was replaced by puromycin resistant gene, then hAID gene with a Flag tag on the N-terminal was cloned into the plasmid between *Hind*III and *Xho*I.

In order to generate the vectors with different enhancers, the Ig enhancer sequence was inserted behind the SV40 polyA sequence (pIg), and then the Ek enhancer sequence was inserted into the *Nru*I site (pIE).

For the pCDNA3.1-GFP, pIg-GFP, and pIE-GFP plasmids, the EGFP gene was inserted into pCDNA3.1/Hygro(+), pIg, and pIE plasmids between *Hind*III and *Xho*I.

To display the anti-HMGB1 full-length antibody (3B1) on the cell surface, the 3B1 light chain with a light chain signal peptide sequence was cloned into the pIE plasmid between *Hind*III and *Xho*I, then hygromycin antibiotic gene was replaced with a neomycin antibiotic gene to generate the pIE-LC plasmid. And the 3B1 heavy chain with a heavy chain signal peptide and a platelet-derived growth factor receptor (PDGFR) transmembrane domain was inserted into the pIE plasmid between *Hind*III and *BamH*I to yield pIE-HC-TM plasmid.

To generate the plasmid PET28a(+)-HMGB1-GFP, EGFP gene was inserted into PET28a(+) between *Sac*I and *Xho*I, and human HMGB1 gene was inserted between *Nco*I and *EcoR*I sites. HMGB1-EGFP can express a fusion protein for monitoring surface-displayed HMGB1 binding antibodies.

### 2.2. Cell Culture

Human non-small cell lung carcinoma cells (H1299, ATCC, Manassas, VA, USA) were maintained in DMEM growth medium with 10% fetal bovine serum (FBS; Minhai Company, Shenzhen, china), 100 U/mL penicillin and 100 µg/mL streptomycin (Gibco, Grand Island, NY, USA). Cells were incubated at 37 °C with 5% CO_2_, and subcultures were carried out every 2–3 days.

293F cells (Invitrogen, Grand Island, NY, USA) were maintained in FreeStyle™ 293 Expression Medium (Invitrogen, USA) in an orbital shaker (BIOTOP, Shanghai, China) at 135 rpm in 37 °C incubator with a humidified atmosphere of 8% CO_2_.

### 2.3. Stable Cell Lines Selection and Mutation Rate Detection

To generate cell clones with stable hAID expression, pPuro-hAID plasmid was transfected into H1299 cells with Lipofectamine 2000 kit (Invitrogen). Two days after transfection, cells were maintained with 5 µg/mL puromycin (Invitrogen) for 10 days. After 10 days selection, cell clones with puromycin resistant were selected and maintained with 1 µg/L puromycin.

In order to select clones with higher hAID-induced hypermutation, the selected clones were seeded into 6-well plate at 2 × 10^5^ cells/well. After 24 h, cells were transfected with a plasmid carrying a mutant GFP gene. Two days after transfection, cells were collected to detect the GFP fluorescence (reverse mutation) by a flow cytometer (FACSCalibur, BD, San Jose, CA, USA). The cell clones with the highest percentage of GFP florescence (H1299-AID-15) were chosen for further study.

To detect the AID-induced mutation rate of GFP on episomal vectors with different enhances. Each of pCDNA3.1-GFP, pCEP4-GFP, pIg-GFP and pIE-GFP plasmids was transfected into H1299-AID-15 cells separately. After transfection, cells were cultured in DMEM medium with 50 µg/mL hygromycin (Gibco, Grand Island, NY, USA) and 1 µg/mL puromycin antibiotics (Gibco, Grand Island, NY, USA) for 15 days. The plasmid and genomic DNA were purified with a DNA purification system (HF206, YuanPingHao Bio, Beijing, China) according to the manufacturer’s protocol. The GFP gene were cloned into pCDNA3.1(+) plasmid and 35–40 clones were randomly picked for sequencing to detect the mutation rate.

### 2.4. Flow Cytometric Analysis and cell Sorting

In this study, FACSAriaIII (BD) and FACSCalibur (BD) flow cytometers were used for analysis and cell sorting, respectively.

To analyze antibody display, the pIE-LC and pIE-HC-TM plasmids were co-transfected in to H1299-AID-15 cells. Two days after transfection cells were collected and incubated with anti-human IgG-APC antibody (Allophycocyanin (APC) conjugated Mouse Anti-Human IgG antibody, BD, 1:20 in cold opti-MEM medium, Invitrogen, USA) for 25 min at 4 °C, then washed with cold PBS once and resuspended with cold opti-MEM, and measured with flow cytometer. The antibody-displaying cells were enriched by flow sorting and cultured with 100 µg/mL G418,50 µg/mL hygromycin and 1 µg/mL puromycin in DMEM medium.

For affinity maturation, the sorted antibody-displaying cells were cultured in DMEM medium with 100 µg/mL G418, 50 µg/mL hygromycin and 1 µg/mL puromycin for about 15 days to obtain more than 10^8^ cells. The cells were collected and stained with APC-conjugated anti-human IgG antibody (BD, 1:20in cold opti-MEM medium) and 10nM HMGB1-GFP antigen in cold opti-MEM medium (Invitrogen, USA) for 30 min at 4 °C. After washing with cold opti-MEM once, the cells were resuspended with cold opti-MEM, and sorted by a FACSAriaIII (BD). The enriched cells were expanded for the next round of affinity maturation.

### 2.5. Antibody Mutants Sequencing and Expression

After affinity maturation, the antibody genes (LC and HC-TM) were cloned into pCDNA3.1/Hygro(+) plasmid (pCDNA3.1-LC and pCDNA3.1-HC-TM) for sequencing to detect antibody mutants.

FreeStyle™ MAX 293 Expression System (Invitrogen, USA) was used to express wild type (WT) and anti-HMGB1 antibody mutants according to the manufacturer’s protocol. Since antibody light chain (LC) and heavy chain (HC) were on different plasmids, various combinations of different LC and HC mutant plasmids were co-transfected into 293F cells with PEI liner 25 kDa (Polysciences, Warrington, PA, USA). After 7 days culture at 37 °C with 8% CO_2_, medium was collected by centrifugation and filtered with 0.45 µm filter membrane, then IgG was purified with protein A affinity chromatography (Invitrogen). Concentrations of purified IgGs were determined with UV spectrophotometer (BioTek, Winooski, VT, USA) and analyzed with SDS-PAGE.

### 2.6. SDS-PAGE and Western Blot

For SDS-PAGE, the antibody expression mediums were analyzed with 12% SDS-PAGE under reducing conditions, followed by Coomassie Brilliant Blue staining.

For Western blot analysis, H1299-AID cells lysates were fractionated under reducing conditions and then transferred onto polyvinylidene difluoride membranes (Amersham Biosciences, Shanghai, China). The AID expression was identified with AID antibody (Cell Signaling Technology, Cat#4975).

### 2.7. Affinity Quantification of Antibody Mutants

Affinities of antibodies were measured by Octet biomolecular interaction machine (ForteBio Octet, Menlo Park, CA, USA). The antibody supernatants were detected by seven Anti-hFc kinetic grade biosensors (ForteBio, 18–5060, Shanghai, China). The detection conditions used were (I) baseline 240 s; (II) loading 240 s; (III) baseline 180 s; (IV) association 120 s with a series of concentrations (1600 nM, 800 nM, 400 nM, 200 nM, 100 nM, 50 nM, 25 nM) of HMGB1-GFP antigen; (V) dissociation 180 s. The K_on_ and K_off_ rates were measured by Octet software and K_D_ was calculated for each antibody mutation by the K_off_/K_on_ ratio.

## 3. Results

### 3.1. Generation of High AID Expression H1299 Cell Clones

Compared with transient transfection, stable expression cell clones have the advantages of stable gene expression at similar levels in each cell. Since a stable cell line could avoid the mutation frequency difference which comes from different AID expressions in cells, we attempted to use stable AID expression cell line to generate AID-induced mutations on target genes. In this work, we employed an AID expression stable cell clone (H1299-AID) to host the episomal vector.

**Figure 1:**
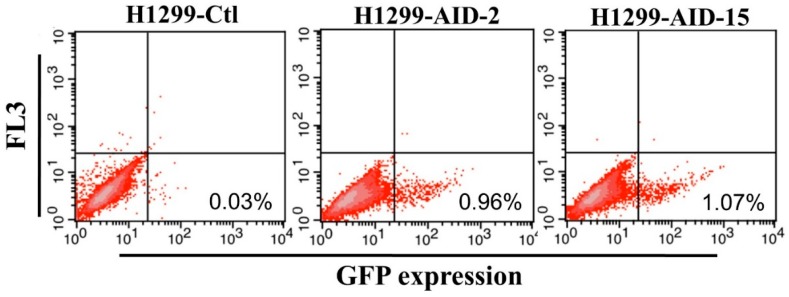
H1299-AID cell clones reverse mutation rate (Clone 2 and Clone 15). H1299-AID clones were transfected with pCEP4-GFPm. 2 days after transfection, the GFP fluorescence (reverse mutation) was detected by flow cytometer. Ctl: negative control. AID: Activation-induced cytidine deaminase expression clones, FL3: FACSAria II FL-3 channel signal results.

In order to generate the H1299-AID clones, the pPuro-hAID plasmid was transfected into H1299 cells, followed by incubation in medium containing puromycin for 10 days. Then, several clones were selected to examine their abilities for somatic hypemutation by transfection of plasmids carrying a GFP mutant (pCEP4-GFPm) into the cells. GFPm has a premature TAG stop codon in the middle of the gene, thus GFPm does not express functional GFP protein. AID can induce reverse mutation at the stop codon to express full-length functional GFP. The efficiency of AID-induced SHM in H1299-AID clones can be measured by detecting GFP positive cells on flow cytometer [47,48]. Twenty puromycin resistant clones were randomly selected to detect the mutation rate. The reverse mutation (GFP positive cells) rate of these clones were around 0.2%~1% (Figure 1, Supplementary Figure S1). The results indicate that AID was successfully expressed in all of these clones. Among these clones, clone 2 (H1299-AID-2) and clone 15 (H1299-AID-15) exhibited ~1% GFP reverse mutation which were higher than all of other clones (<0.75%). (Supplementary Figure S1). AID expressions were further confirmed by Western blot (Supplementary Figure S2), and the transcription level of AID in clone 15 was much higher than that in clone 2. Clone 15 had the highest GFP fluorescence, GFP reverse mutation rate (1.07%) (Figure 1) and AID expression. Therefore, this clone was chosen as the host cell clone for further experiments.

### 3.2. Enhancers Increased AID-Induced Hypermutation on Episomal Vectors

**Figure 2:**
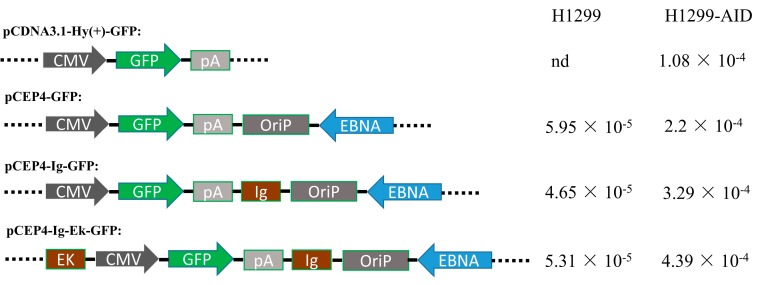
Structure of the plasmids with or without enhancers and mutation rate (mutation rate/15 days) in H1299 and H1299-AID cells. CMV: Human cytomegalovirus immediate-early promoter/enhancer. GFP: green fluorescent protein. pA: SV40 polyadenylation signal. OriP: EBV origin of replication. EBNA: Epstein-Barr nuclear antigen. EK: Ek enhancer sequence. lg: Ig enhancer sequence.

The Ig and Ek enhancers were inserted into a pCEP4-GFP plasmid to generate plasmids with one (pIg-GFP) or two enhancers (pIE-GFP) (Figure 2). The generated episomal and chromosome-integrating pCDNA3.1-GFP vectors (Figure 2) were transfected into H1299 and H1299-AID cells, separately. Transfected cells were maintained in medium containing hygromycin and puromycin antibiotics for 15 days. The GFP gene was then cloned out and sequenced to detect the mutation rate. As shown in Figure 2, no background mutations were detected in pCDNA3.1-GFP plasmid, and around 5 × 10^−5^ background mutation rate was obtained in episomal vectors in H1299 cells. While in H1299-AID cells, 1.08 × 10^−4^ mutation rate was obtained in pCDNA3.1-GFP plasmid and 2.20 × 10^−4^ mutation rate was detected in pCEP4-GFP episomal vector. The pCEP4-GFP episomal vector had higher mutation rate than pCDN3.1- GFP in both H1299 and H1299-AID cells. This indicated that the episomal vector was more efficient to accumulate mutations than random-integrated plasmid (pCDNA3.1-GFP). Compared with the original pCEP4-GFP vector, higher mutation rate was detected in the enhancers-containing plasmids (pIg-GFP, 3.29 × 10^−4^; pIE-GFP, 4.39 × 10^−4^) in H1299-AID cells. The highest mutation rate was detected in the plasmid containing two enhancers, which had a two-fold mutation rate compared to that of the original pCEP4-GFP plasmid. Higher mutation rates in the enhancer-containing vectors indicate that the enhancers increased AID-induced SHM on the pCEP4 vector.

### 3.3. Anti-HMGB1 Full-Length Antibody Display and Affinity Maturation

In order to display antibody on H1299-AID cell surface, the 3B1 antibody light chain (LC) and heavy chain (HC) with a PDGFR trans-membrane domain (HC-TM) were cloned into two episomal vectors containing two enhancers to generated light chain (pIE-LC) and heavy chain (pIE-HC-TM) expression plasmids (Supplementary Figure S3A). By co-transfection of the two plasmids into H1299-AID cells, the antibody was successfully displayed on the cell surface (Supplementary Figure S3B, Region R1).

**Figure 3:**
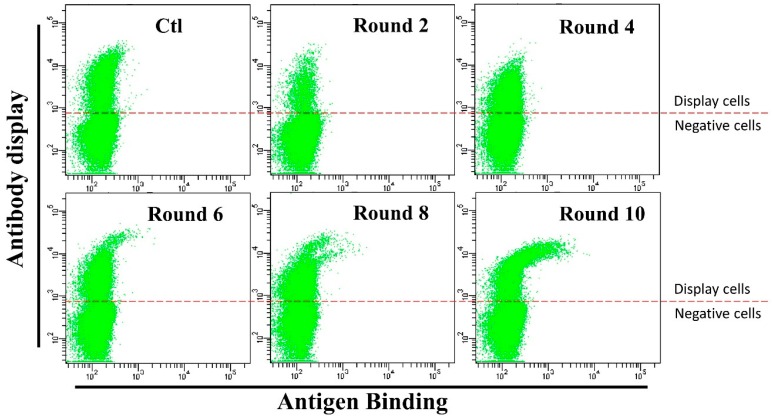
Flow cytometry cell sorting results during affinity maturation. Each round of maturation, the 3B1 antibody on the cells were stained with anti-human-IgG-APC (1:20 dilution) and HMGB1-GFP (0.5 µg/mL). Cells with better HMGB1-GFP binding (0.05–0.1% of the cells) were sorted out and expanded for another round of sorting. Anti-human-IgG-APC: Allophycocyanin conjugated Mouse Anti-Human IgG antibody. HMGB1-GFP: High mobility group box 1 and GFP fusion protein.

Cells displaying 3B1 were then harvested by cell sorting using a flow cytometer and maintained in medium containing G418 and hygromycin for affinity maturation. In each round of affinity maturation, 10^8^ cells were co-stained with anti-human IgG-APC antibody and HMGB1-GFP fusion protein. Then, the cells displaying the best binders (0.05–0.1% cells with highest antibody binding/antibody display ratio) were enriched by flow sorting and expanded for subsequent round of affinity maturation. Ten rounds of affinity maturation were performed for the affinity maturation of this antibody (Figure 3). After six rounds of maturation, cells with significantly higher HMGB1-GFP binding efficiencies appeared (Cells in Figure 3). After eight rounds, higher antigen binding cells consisted of two different populations (Figure 3). This result suggests that different mutants were enriched during affinity maturation. Antibody LC and HC-TM genes from rounds six, eight and ten sorted cells were cloned into the pCDNA3.1(+) plasmid and sequenced. As shown in Table 1, two light chain (D196Y, H218Y) and four heavy chain (L32V, S52N, S98R, and F176L) amino acid mutations were detected. The light chain with H218Y and heavy chain with S52N and F176L amino acid mutations were enriched during affinity maturation process.

H218Y light chain mutation was first detected in round 6 (H218Y: 3/46), and enriched in round 8 (H218Y: 9/39) and round 10 (H218Y: 14/51, D196Y + H218Y: 1/51). The S52N and F176L heavy chain mutations were first detected in round 8 (F176L: 2/43, S52N + F176L: 24/43). All the sequenced heavy chains had both S52N and F176L amino acid mutations except one clone which has only S52N mutation (S52N + F176L:54/55, S52N: 1/55) in round 10. While other detected amino acid mutations only showed once in all of the sequenced plasmids, indicating that AID functions through the whole maturation process. The AID expression of round 4,6, 8, 10 cells were convinced by Western blotting (supplementary Figure S4). No significant AID expression lost during the affinity maturation process.

### 3.4. Antigen Binding and Affinity Determination

**Figure 4:**
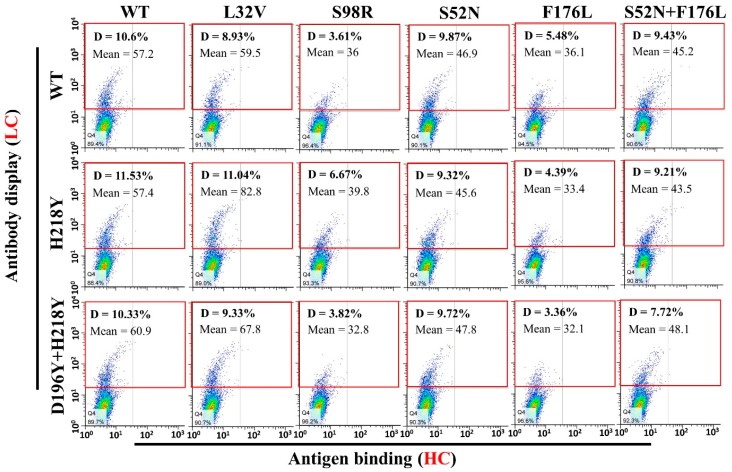
Antibody mutants display on cell surface and binding with HMGB1-GFP. Various antibody light chain and heavy chain mutants with trans-membrane domain were co-transfected into H1299 cells. Two days after transfection, cells were stained with anti-human-IgG-APC and HMGB1-GFP. Antibody display was evaluated by flow cytometry (Gate D: Positive Cells). The heavy chain S98R and F176L mutant combinations groups have much fewer cells display the antibody on the cell surface (Gate D, S98R: 3.6–6.6%, F176L: 3.35–5.45%) than other heavy chain mutant combinations (Gate D, 7.7–11.5%). WT: Original wild type antibody.

**Figure 5:**
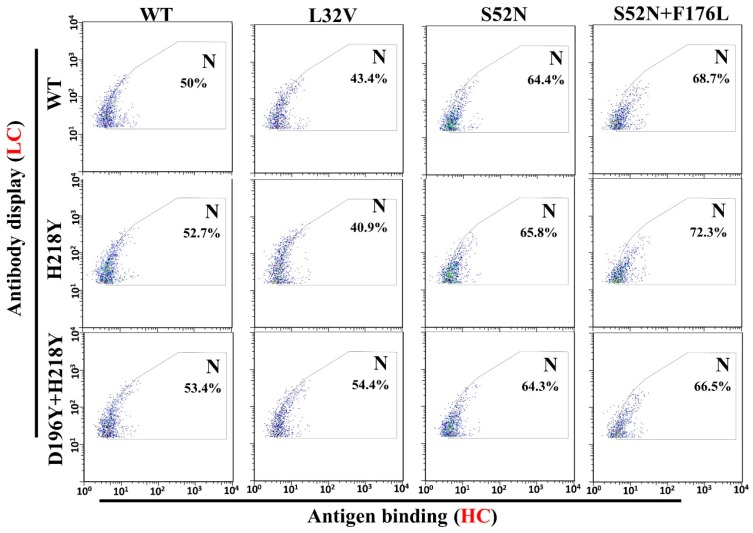
Statistics of HMGB1-GFP binding with mutant combinations (well displayed combinations). Gate N was chosen according to the center of the WT/WT cells, and half of the cell were in the gate. Compared with the WT/WT, combinations with better antigen binding will have higher percentage of cells in gate N. According to the results, light chain H218Y, D196Y + H218Y, and heavy chain L32V mutants have no significant contribution to antigen binding (Lower or similar percentage of cells in gate N compared with WT/WT). While the heavy chain S52N and S52N + F176L mutants significantly improved the antigen binding (higher percentage of cell in gate N).

To evaluate antigen binding of various LC and HC mutant combinations, pCDNA3.1-LC and pCDNA3.1-HC-TM were co-transfected into H1299 cells. Two days after transfection, antigen binding of these mutant combinations were subject to flow cytometry. All the antibody combinations successfully displayed on the cell surface (Figure 4, Gate D). However, a fewer percentage of cells displayed the heavy chain S98R and F176L mutant combinations (Figure 4, Gate D. S98R combinations: 3.61–6.67%, F176L combinations: 3.36–5.48%, Other combinations: 7.72–11.53%). Meanwhile, the quantity of S98R and F176L mutant combinations on the cell surface also lower than other combinations (Figure 4, Gate D, lower Mean of the antibody display cells). Therefore, the well displayed combinations were compared with the original WT/WT (LC/HC) for the antigen binding (Figure 5, Gate N). The light chain H218Y, D196Y + H218Y, and heavy chain L32V mutant combinations have no significant improvement of antigen binding (Figure 5, Gate N. WT/WT = 50%, H218Y/WT = 52.7%, D196Y + H218Y = 53.4%, WT/L32V = 43.4%). While stronger bindings were detected in heavy chain S52N and S52N + F176L mutant combinations (Figure 5, Gate N. S52N combinations: 64.3–65.8%, S52N + F176L: 66.5–72.3%). The heavy chain S52N and S52N + F176L mutants were good candidates for expression to detect affinity.

According to the enrichment of different mutants, the light chain H218Y were enriched during the affinity maturation (Table 1). Therefore, the heavy chain S52N, S52N + F176L and light chain H218Y mutants were chosen for expression to detect the affinity with surface plasmon resonance (SPR). As shown in Table 2, all the chosen mutant combinations have higher affinities than WT antibody. In the light chain H218Y mutant group, the H218Y/WT combination showed around 3.6-fold affinity improvement than WT/WT, and more amino acid mutation in the combinations showed higher affinity (WT/WT < H218Y/WT < H218Y/S52N < H218Y/S52N + F176L) (Table 2). In the light chain WT group, the WT/S52N + F176L combination have more than 10-fold affinity improvement than the WT antibody (WT/WT). However, the highest affinity was detected in WT/S25N combination which has about 20-fold affinity improvement compared to WT antibody (WT/WT (4.47 × 10^−8^), WT/S52N (2.3 × 10^−9^)) (Table 2). Since the heavy chain S52N + F176L mutant was detected in round 8 and enriched in round 10 (54 out of 55 sequenced clones were this mutant), and the S52N mutant was detected only once in round 10. The S52 may come from S52N + F176L reverse mutation. The antibody expressions were determined by SDS-PAGE and the chosen combinations had similar or higher expression than WT (Supplementary Figure S5).

## 4. Discussion

Compared with microbe-based display technologies, mammalian cell-based display has advantages in pre-eminent expression, stability, folding, modification, and codon usage [14,15,16,49]. However, it is severely flawed in library size and library stability. To develop a stable library in mammalian cells, chromosome-integrating [17] or episomal vectors [16,18] can be used for gene expression. Chromosome-integrated plasmids will randomly insert into the cell genome to develop stable cell clones while episomal vectors can maintain in mammalian cell as episomes [50,51]. Because of the low efficiency of random integration, it is hard to develop a library ≥10^6^ by inserting antibody gene expression cassette into the cell genome. Episomal vectors do not integrate into cell genome. However, transient transfection will lead to multiple antibody genes in a single cell. Theoretically, if there are two different antibodies in one cell, 10 kinds of IgGs can be expressed and displayed on the cell surface [52]. Cells with more than two different antibody genes may express tens or hundreds of IgGs. So, multiple antibody genes in the cell will seriously affect the quality of the antibody library and subsequently antibody selection. In order to eliminate multi-copy antibody genes being transfected into target cells, low transfection efficiency is needed [16]. Meanwhile, like the integrate plasmids, antibiotic selection is also needed to generate stable antibody expression cells after transfection. All of these drawbacks make it difficult to develop an ideal library in mammalian cells.

Because of the low efficiency associated with generating antibody libraries using random integration or episomal vectors, activation-induced cytidine deaminase (AID) was chosen to generate antibody libraries for antibody affinity maturation [27,28]. During cell proliferation, AID induces mutations in the antibody gene to develop a mutation library, and the cells containing better antigen binding mutants can be harvested by flow sorting. However, the efficiency of these systems has been greatly restricted by low mutation rate (≤10^−5^ bp per generation) of AID-induced SHM in vitro [12,23].

Several different methods can be used to improve the efficiency of AID-induced SHM in vitro. Since higher AID expression will increase the chance of AID binding with the target genes to induce mutations [53,54], selection of cell clones with high AID expression in the host cell line will be a prudent choice. In the past years, many efforts have been put to select AID mutants (AIDm) with higher activity in different cell lines [55,56,57,58]. Compared with the intensive labor put into AID protein engineering, we sought to enhance AID-associated SHM by inserting enhancers into the DNA sequence near the target gene, because of the rationale that enhancers could greatly enhance the frequency of SHM to nearby transcribed genes [34,35,36,37,38].

In this work, we selected a stable AID expression cell clone which has a 1.07% GFPm reverse mutation rate as the SHM host cell line. Meanwhile, two different enhancers were chosen to improve AID-induced SHM to our targeted genes (GFP and antibody) on pCEP4 episomal vectors. The enhancers (Ig and Ek enhancers) were inserted into pCEP4 episomal vector to generate plasmids with one or two enhancers. Then the generated episomal and chromosome-integrating plasmids were transfected into H1299 and H1299-AID cells separately to detect the mutation rate of the target GFP gene on these plasmids. By comparing the GFP mutation rates of different plasmids in H1299 and H1299-AID cells, we found that the episomal vector (pCEP4-GFP, 2.2 × 10^−4^/15 days) has a two-fold higher mutation rate than chromosome-integrating plasmids (pCDNA3.1-GFP, 1.08 × 10^−4^/15 days) in H1299-AID cells. This indicated that the episomal vector was more efficient in accumulating mutations than chromosome-integrating vectors. We also found that vectors with enhancers in the backbone had higher AID-induced SHM than the original pCEP4 vector in H1299-AID cells. The plasmid with two enhancers had much higher mutation rate than the plasmid without or with only one enhancer (Figure 2), suggesting that more enhancers in the plasmid could improve the SHM on episomal vectors. This offers us a way to improve the AID-induced SHM to target gene by adding more enhancers in the vector.

With the engineered episomal plasmids, we displayed an anti-HMGB1 antibody on the H1299-AID cell surface. After ten rounds of sorting, three light chain (D196Y, H218Y, D196Y + H218Y) and five heavy chain (L32V, S52N, S98R, F176L, and S52N + F176L) mutants were detected. The light chain H218Y and heavy chain S52N, S52N + F176L amino acid mutations were chosen for expression and the affinity of these antibodies were determined by SPR. According to the affinity results, all the combinations have higher affinity than the WT/WT. Interestingly, the H218Y mutant which contains a Histidine mutated to Tyrosine at the light chain 218 constant region showed around 3.6-fold affinity improvement. This result indicates that the constant region also contribution to antibody affinity. In general, more amino acids mutations in light chain and heavy chain means higher affinity (LC/HC: WT/WT (4.47 × 10^−8^) < H218Y/WT (1.24 × 10^−8^) < H218Y/S52N (8.56 × 10^−9^) < H218Y/S52N + F176L (4.59 × 10^−9^) and WT/WT (4.47 × 10^−8^) < WT/S52N + F176L (5.11 × 10^−9^) (Table 2). Except one mutant which only has S52N amino acid mutation in the heavy chain, which showed almost 20-fold affinity improvement than the WT (WT/WT (4.47 × 10^−8^), WT/S52N (2.30 × 10^−9^)).

During the affinity maturation process, the first four rounds of affinity maturation showed no significant improvement of antigen binding from flow cytometry results, while rounds six, eight, and ten yielded cells of much higher antigen binding (higher antibody binding/antibody display ratio cells in FACS results, Figure 3). Low efficiency in accumulating high affinity antibody mutants at the beginning was probably because of multiple antibody genes in the cells. As we have mentioned before, multi-copy antibody genes in a single cell seriously affect the efficiency of affinity maturation. After a long duration of cultivation, a large fraction of cells was lost in antibody plasmids and maintained low copy number of antibody vectors in the cell, thus antibody mutant clones were detected after 6 round maturation.

In microbe antibody affinity maturation systems (such as phage display, yeast display, etc.), error-prone PCR was widely used to generation antibody mutation libraries [59,60]. Mutation rates from 1% to 27% were chosen by different groups to develop libraries [61,62]. Due to the lack of proper modification, peptide folding and the hypermutation effect, mutants selected from these microbe libraries may cause detrimental effects for therapy. While a mammalian cell has the advantages of protein expression, more advanced peptide folding and post-translational modification, mutants generated from mammalian cell platforms may be used for therapeutic purposes

Since enhancers also works in the cell genome, placing enhancers next to the antibody gene may be a good choice for the generation of stable cell clones with high antibody expression for affinity maturation.

## Figures and Tables

**Table 1:** Antibody mutants.

Mutants Rounds	Light Chain (Mut/Seq) ^1^	Heavy Chain (Mut/Seq)
Round 6	H218Y (3/46)	S98R (1/41) L32V (1/41)
Round 8	H218Y (9/39)	F176L (2/43) S52N + F176L (24/43)
Round 10	H218Y (14/51) D196Y + H218Y (1/51)	S52N (1/55) S52N + F176L (54/55)

^1^ Detected mutation clones (Mut)/sequenced clones (Seq).

**Table 2:** Affinity of different mutants.

Mutants (LC/HC ^1^)	K_on_ (1/Ms)	K_off_ (1/s)	K_D_ (M)
WT/WT	5.18 × 10^4^	2.32 × 10^−3^	4.47 × 10^−8^
WT/S52N	3.90 × 10^5^	8.96 × 10^−4^	2.30 × 10^−9^
WT/S52N + F176L	1.72 × 10^5^	8.81 × 10^−4^	5.11 × 10^−9^
H218Y/WT	1.09 × 10^5^	1.36 × 10^−3^	1.24 × 10^−8^
H218Y/S52N	1.55 × 10^5^	1.33 × 10^−3^	8.56 × 10^−9^
H218Y/S52N + F176L	2.08 × 10^5^	9.55 × 10^−4^	4.59 × 10^−9^

^1^ antibody light chain and heavy chain mutant combinations.

## Supplementary Materials

The following is available online at http://www.mdpi.com/2073-4468/7/4/42/s1. Figure S1: The GFP reverse mutation of 20 different H1299-AID clones. Figure S2: Western bolt of AID expression in H1299-AID cells (clone 2 and clone 15). Clone 15 has higher AID expression than clone 2. GAPDH: Glyceraldehyde-3-phosphate dehydrogenase which constitutively expressed in H1299 cell as loading control. AID: Activation-induced cytidine deaminase. Figure S3: Antibody display plasmids maps and the FACS results of 3B1display on H1299-AID cells. A. Antibody light chain and heavy chain plasmids maps for antibody display. B. Antibody display results. Light chain and heavy chain (with trans-membrane domain) plasmids were co-transfected into H1299 cells. 2 Days after transfection, cells were stained with APC-conjugated anti-human IgG antibody (BD, 1:20) and detected with FACS. The 3B1 IgG was successfully displayed on the H1299-AID cell surface (Region R1). Ctl: Empty vector control. 3B1-IgG: 3B1 light and heavy chain plasmids transfected cells. FL1: FACSAria II FL-1 channel signal results. Figure S4: Western bolt of AID expression in the sorted cells during affinity maturation process (Round 4, 6, 8 and 10). Figure S5: SDS-page results of different 3B1 light chain and heavy chain mutants’ combination productivity in 293F cells. All the combinations have similar or higher expression than the WT antibody. BSA: Bovine serum albumin band. 3B1-HC: 3B1 IgG heavy chain band. 3B1-LC: 3B1 IgG light chain band.
