# Supplementary material for: Enhancers Improve the AID-Induced Hypermutation in Episomal Vector for Antibody Affinity Maturation in Mammalian Cell Display
Source: Antibodies (Basel). 2018 Dec 13;7(4):42. doi: 10.3390/antib7040042 (PMC6698961; doi:10.3390/antib7040042)
Supplement: Supplementary file 1 [file antibodies-07-00042-s001.pdf]

antibodies

Supplementary Materials:

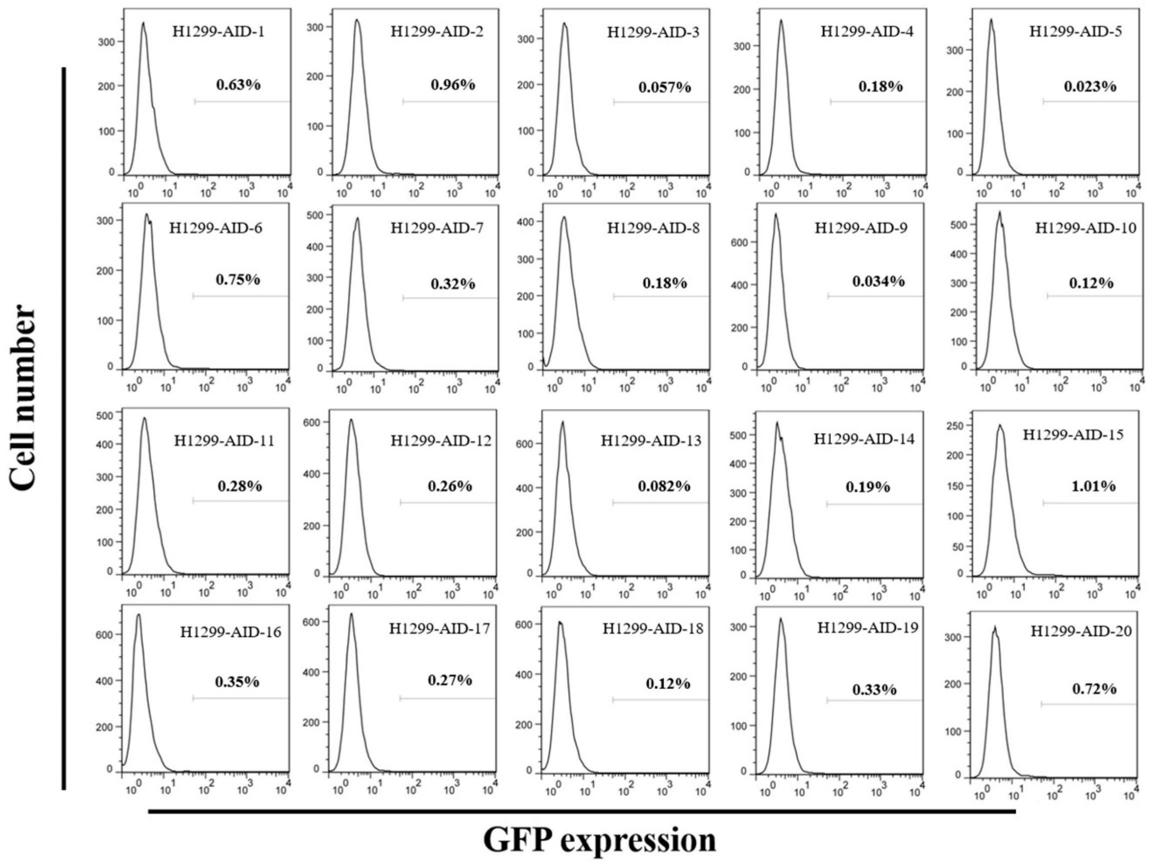

Figure S1. The GFP reverse mutation of 20 different H1299-AID clones

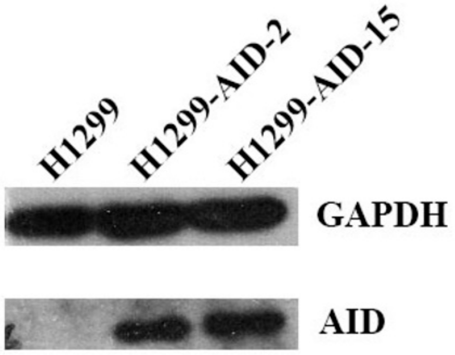

Figure S2. Western bolt of AID expression in H1299-AID cells (clone 2 and clone 15). Clone 15 has higher AID expression than clone 2. GAPDH: Glyceraldehyde-3-phosphate dehydrogenase which constitutively expressed in H1299 cell as loading control. AID: Activation-induced cytidine deaminase

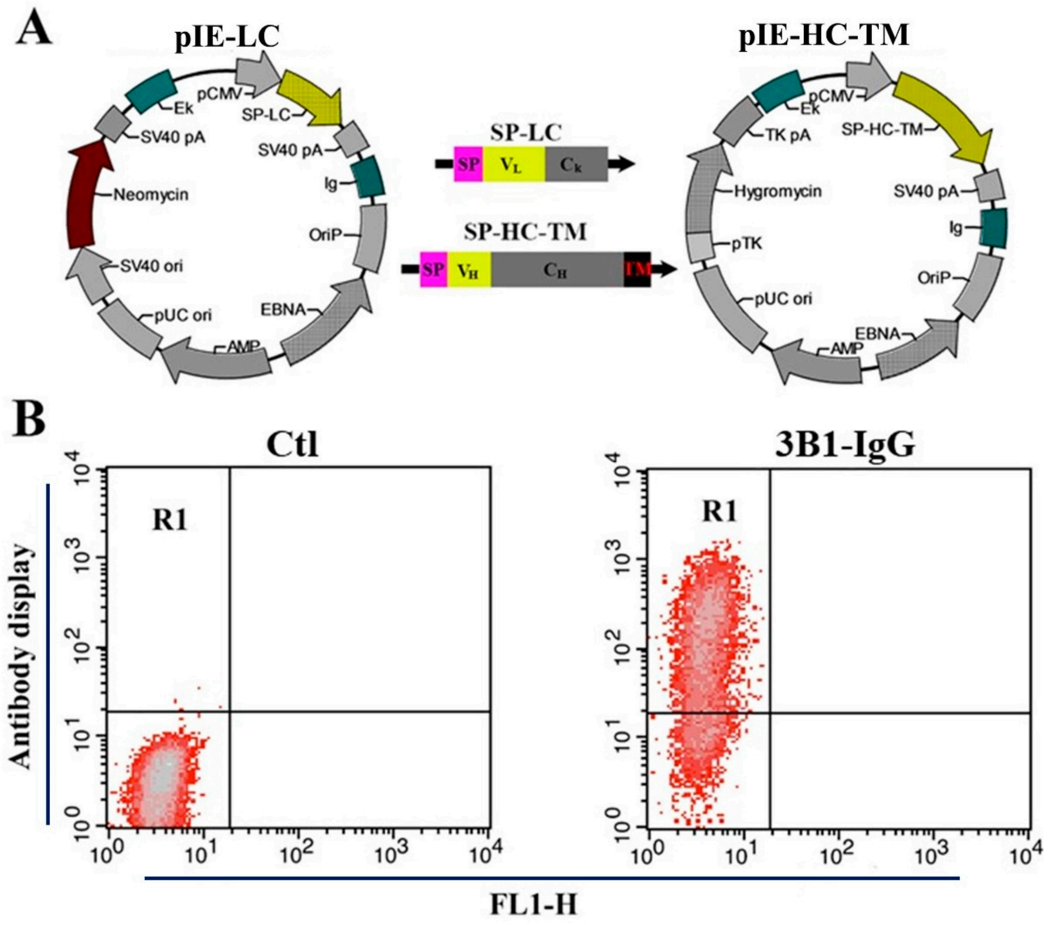

Figure S3. Antibody display plasmids maps and the FACS results of 3B1display on H1299-AID cells. **A**. Antibody light chain and heavy chain plasmids maps for antibody display. **B**. Antibody display results. Light chain and heavy chain (with trans-membrane domain) plasmids were co-transfected into H1299 cells. 2 Days after transfection, cells were stained with APC-conjugated anti-human IgG antibody (BD, 1:20) and detected with FACS. The 3B1 IgG was successfully displayed on the H1299-AID cell surface (Region R1). Ctl: Empty vector control. 3B1-IgG: 3B1 light and heavy chain plasmids transfected cells. FL1: FACSAria II FL-1 channel signal results

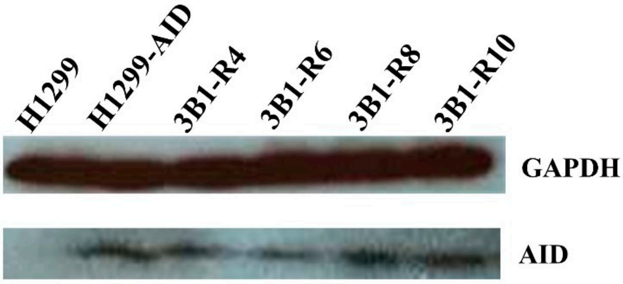

Figure S4. Western bolt of AID expression in the sorted cells during affinity maturation process (Round 4, 6, 8 and 10).

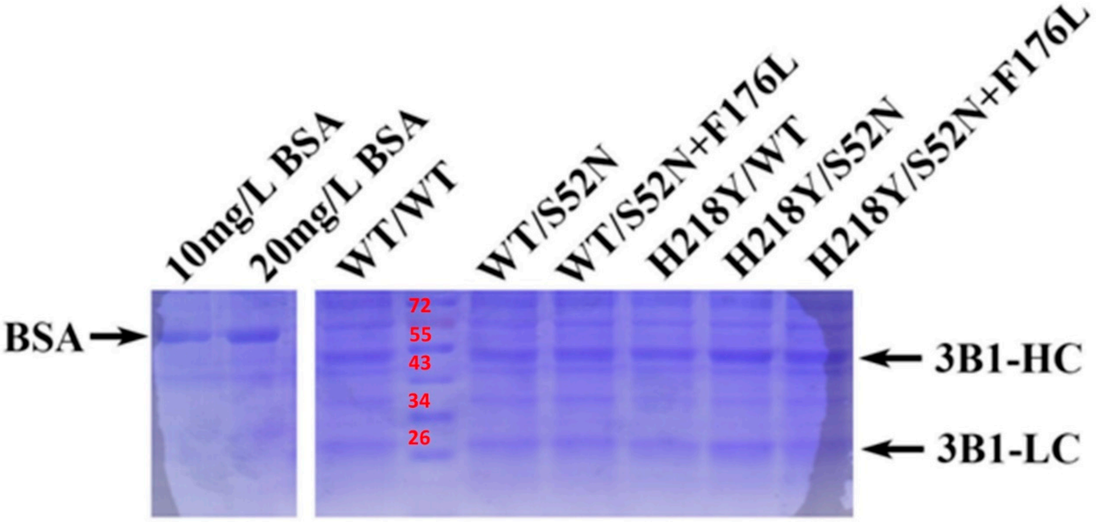

Figure S5. SDS-page results of different 3B1 light chain and heavy chain mutants' combination productivity in 293F cells. All the combinations have similar or higher expression than the WT antibody. BSA: Bovine serum albumin band. 3B1-HC: 3B1 IgG heavy chain band. 3B1-LC: 3B1 IgG light chain band.
